# Surgical Observations on the Restoration of the Nose; and on the Removal of Polypi and Other Tumours from the Nostrils. From the German of Dr. Diffenbach, of Berlin. With the History and Physiology of Rhinoplastic Operations, Notes, and Additional Cases

**Published:** 1834-01-01

**Authors:** 


*Surgical Observations on the Restoration of the Nose; and on the Removal of Polypi and other Tumours from the Nostrils. From the German of* Dr. Dieffenbach, *of Berlin. With the History and Physiology of Rhinoplastic Operations, Notes, and additional Cases.* By John Stevenson Bushnan, f.l.s., m.r.c.s. e., &c.—8vo. pp. 159. London, 1833.

A review of the first volume of Dr. Dieffenbach's work, in the Edinburgh Medical and Surgical Journal, induced Mr. Bushnan to undertake the translation of the portion relating to the removal of tumours from the nose, and its restoration, when destroyed or dilapidated. Although the mode of performing these operations is tolerably well understood, they are not so felicitously executed as to make it supererogatory to attempt to improve them. Such attempts have been more fortunate in the hands of continental than of English surgeons; and this it may be presumed was the translator's inducement, as it is certainly his warrant. Many cases of Rhinoplastic operations are before the profession, yet our information is necessarily incomplete without reference to the practice of foreign practitioners. There are perhaps few subjects of equal importance which have engaged the attention of so small a portion of the profession. Even the title is generally misunderstood, and surgeons have actually published cases in which the operations were described as Taliacotian; ignorant of the fact that the process of Tagliacozzi was essentially different from the operations they had performed themselves, and most potently believing that he was the discoverer of an operation that there is abundant proof was not unknown to Galen, Paul of Ægina, Fabricius ab Aquapendente, Celsus, and others. Nor do they for a moment distrust the statement of the witty immortality that Butler has conferred upon Tagliacozzi, by asserting that the Italian cut supplemental noses from the anonymous extremities of compatriot porters; ignorant that the operation now adopted throughout Europe, Asia, and America, is the invention of the Brahmins of India.

The date of the operation is unknown, for it has been performed in India from time immemorial. Mr. Bushnan conjectures that the first public account appeared in the Gentleman's Magazine for 1794. One of the maxims of political economy (to some paradoxical) is here oddly enough illustrated; namely, "that the demand creates the supply." Throughout the East, where despotism in all its modes holds dominion, one of the favourite caprices of cruelty is mutilation. As the following example is only one of many, it is easy to conceive that the demand for noses would be to an extent to encourage their production; and as monopolies are not peculiar to periods, but have extended through all time since men discovered that to plunder is easier than to work, and that wholesale robbery is better than stealing in detail it naturally followed that the manufacture of noses and lips became the privilege of a caste of Hindoos called Koomas.

"We may form some idea of the horrible extent to which the amputation of noses and lips was formerly carried in India, and the frequency and urgency therefore of the calls upon human ingenuity to repair the loss so sustained, by the following narrative related by Father Guiseppe, of a fact which occurred no longer ago than the year 1769 or 1770. 'The city of Kirtipoor, in Nepaul, being besieged by the Ghoorka army, was betrayed by one of its nobles. The inhabitants might still have stood on their defence; but, on the promise of amnesty, they surrendered themselves prisoners. Two days afterwards, Pritwinarayan, the king of Ghoorka, their conqueror, ordered the principal persons of the town to be put to death, and the lips and noses of every one, even the infants who were not found in their mother's arms, to be cut off; directing at the same time that the lips and noses should be preserved, that he might ascertain the number of souls, and that the name of the town should be changed into *Nascatapoor*, which signifies (such relationships have the languages,) *Nose-cut-town*. The order was carried into execution with every mark of horror and cruelty, none escaping but those who could play upon wind instruments. Many of them, in despair, put an end to their lives; others came to us, in great bodies, in search of medicines; and it was most shocking to see so many living people with their teeth and noses resembling the skulls of the dead." (P. 30.)

The first chapter is occupied with the history of the operation, and abounds with references to authorities, not without interest, although of questionable utility to any but the curious, who delectate in leisure denied to the labouring practitioner. Mr. Bushnan's physiology in relation to the operation (and in this he revels,) is sufficiently orthodox, and always ingenious, and contains a view, which, although neither new nor peculiar to himself, is striking, and will have the interest of novelty to some who have been embarrassed by the circumstance of parts of adoption taking on the function and properties of the structure to which they were before aliens. If we keep in mind the processes always in antagonism, yet in health always in equilibrium, absorption, and reparation, we shall do justice to the following satisfactory solution.

"With respect to the process by which mucous membranes, when brought to the surface of the body, take on the character of common integuments, and the latter, when made to line canals or cavities, assume that of mucous membranes, very little can be said, so long as we continue in almost total ignorance of the nature of absorption and secretion, and of the other molecular actions of the living body. It is a singular circumstance that, in the process of healing by the first intention, whatever be the character of the tissues to be united, no difference whatever can be perceived in that of the lymph which is to become the medium of this reunion, and which is ultimately to take on the character of the tissues in question; and it is not improbable that the lymph which is deposited in the nidus of even the morbid tissues, such as a tubercle or a scirrhus, is of precisely the same character as that which is instrumental in renewing mucous or dermoid membranes. The character of the tissue which the lymph, in any case, ultimately assumes, seems to depend upon the specific action of the vessels by which it is organized; which specific action is the result partly of the peculiar kind of irritability with which the capillary vessels of every individual organ of the body appear to be endowed, and partly by the peculiar kind of stimuli to which these capillary vessels are everywhere exposed; and if it is these causes which determine the character of any tissue as formed *de novo*, it must be to similar causes that we should look for an explanation of the changes in character which any tissue is liable subsequently to undergo. So long as the irritability of any two secreting vessels, and the stimuli by which this irritability is excited are the same, the secretions from each, it much be presumed, will be identical; but, if either of these conditions be different, the secreted matters will differ in proportion. It is in this way alone that we can explain why the capillary vessels of the secreting surfaces of the uterus, the mammæ, the kidneys, and so on, acting upon precisely the same kind of blood, in their natural state, should form from it respectively perfectly distinctive fluids; and why those of the mucous, dermoid, serous, and other tissues, should, in their natural state, deposit each its own peculiar tissue, and no other. But it is abundantly well known that, under peculiar circumstances, each of the former sets of vessels is capable of secreting fluids not naturally its own, but more or less allied to the natural secretions of others. Thus, numerous cases are on record in which the menstrual fluid, or something very like it, has been discharged from the nostrils, eyes, ears, mouth, lungs, stomach, anus, mammæ, navel, skin, and other organs; others, in which the milk, or a similar fluid, has been passed from the eyes, mouth, urinary bladder, vagina, and navel; and others, in which the urine, or at least many of its characteristic principles, have been voided by the nostrils, eyes, ears, stomach, mammæ, navel, and skin.* Now, the continued renewal of the

* Schenck, Bartholin, Schulrig, Mollenbroech, Percival; Edinburgh Med. Commentaries. Halford; Med. Trans., 1820. Coindet; Inaug. Dissert., 1820. Dumas and Prevost, Annales de Chemie, 1823. Mayer; Zeitschrift fur Phys., 1827. Arnold; American Journ. of Med., 1827, &c.

particles of each of the solid tissues from the blood, in proportion as they are removed by absorption, is by a process of secretion precisely similar to that by which the several fluids are deposited: and if vessels naturally engaged in secreting, for example, mucilage alone, can, under peculiar circumstances, secrete the menstrual fluid, milk, or urine, it ought not to surprise us that vessels, the natural office of which is to deposit particles of mucous membranes, should, in certain cases, deposit particles of common integument, or *vice versâ;* particularly when we consider that there is infinitely less dissimilarity in the structure of these two tissues than in the character of many of the fluids which are occasionally vicarious of each other. If, then, the mucous surface of a portion of lip, reflected, as in the operation introduced by Mr. Liston for forming a new columna nasi; or a portion of the lining membrane of the mouth, drawn from this cavity and laid over the cheek, as in an operation of Dieffenbach for relieving a contracted mouth, soon acquires all the properties of the dermoid tissue, on the one hand; and if, on the other, the dermoid surface of the lip, as in the operation for forming the columna, or of a strip of the integuments of the perineum, as in Mr. Earle's introduced method of removing a gap in the urethra; or lastly, of the prepuce, reflected inwards, and kept thus in contact with the glans penis, as in another operation of Dieffenbach, sooner or later degenerates into a perfect mucous membrane; it is not calculated, I conceive, to excite surprise, or more than we may reasonably attribute to the very different circumstances in which the respective tissues are now placed, and the very different stimuli to which their capillary vessels are now exposed. In proportion as the particles of the original tissue are absorbed, particles of a tissue somewhat different will be deposited; and what was originally a thin, semi-transparent, spongy membrane, with mucous follicles on one surface, and a viscid fluid on the other, will gradually pass into a thick, opaque, dense substance, with sebaceous follicles below and cuticle above, or *vice versâ.* It is by this admirable provision that nature has enabled us to meet so many emergencies, against which the art of man would otherwise have contended in vain; by placing within each organ a power, not only of repairing itself *en masse*, when divided or otherwise injured, but of adapting itself, particle by particle, to the innumerable varieties and circumstances to which it is liable to be exposed, and under which, if destitute of this power, it must necessarily have been speedily destroyed." (P. 44.)

The difference between the Indian operation (for so we shall continue to speak of it,) and that of Tagliacozzi, principally consists in the *source* from whence the exotic organ is derived; the superior advantage of inosculating from a neighbouring region rather than a remote one, is so obvious, that the marvel is that the Italian process should have been thought of without immediate rejection. Compare the convenience of transplanting the portion of structure required to supply the deficiency from the immediate vicinity of the ruin, with the painful and irksome discipline of securing for many days the arm to the head, while the alien integument was becoming a denizen of the face. However, after conceding the palm to the Oriental practice in this particular, Dr. Dieffenbach takes some grave, and not unfounded exceptions: he contends, that one result is, often, unenviable deformity in the part of the face or forehead furnishing the graft; another, the inferior beauty of the new edifice to the one which can be raised by the modification of the Brahminical operation recommended by himself. He describes his method of restoring " depressed noses, as the simplest, most natural, and the easiest of accomplishment, of any yet proposed." These are his words:

" *Operation.* The method I follow in operating for restoring depressed noses, is unquestionably the simplest, the most natural, and the easiest of accomplishment of any yet proposed. It simply consists in dividing into portions the remains of the old and now sunken organ, raising them from the cavity in which they lie, securing them by appropriate fastenings, and maintaining them during the process of healing, by proper means of support, in the position they formerly occupied. To explain this more fully, I shall enter into a detailed description of the operation. The patient being seated on a chair, behind which an assistant holds the head firmly against his breast, the operator thrusts a small-pointed scalpel into the left side of the cavity before the sunken point of the nose, and, by an incision proceeding obliquely upwards, cuts through the soft parts as far as the nasal process of the frontal bone. A similar incision is then made on the right side. The strip of integuments between these incisions, which consists of the point and dorsum of the old nose, is twice as broad below as above, where it is attached to the skin of the forehead. At the lower end it is connected with the upper lip only by the shrunken cutaneous portion of the septum. If this be destroyed, and in order to gain room, the flap may at once be raised and reflected. The inverted tip of the nose, which forms the extreme point of the flap, is next to be pressed outwards, and the shortened septum is to be elongated by an incision made on each side of it in the upper lip.

" The next step is the formation of the sides of the nose. The first incision is most conveniently made on the right side of the face: the knife is inserted down to the bone, a few lines below the termination of the incision by which the dorsum of the nose was raised; it is then to be carried slowly through the soft parts, obliquely downwards on a line where the base of the nose passes into the integuments of the cheek; a small addition from the latter being advantageous. A similar incision is then to be made on the left side.

"Lastly, two semicircular incisions are to be made through the soft parts, along the natural (though in the present instance entirely obliterated) place of insertion of the alæ nasi. Both the left and right of these incisions pass round the lower part of the sunken alæ, outwards and upwards, meeting the incisions at the sides of the nose. The lower edges of these flaps are now partially free; they are to be laid hold of with the forceps, and cautiously separated from the bones, and then with their alæ, situated at their lower part, are to be drawn out of the cavity, and reflected upwards. The flaps extend to the skin of the forehead, as they approach which they become narrower.

"The subsequent step of the operation, and a very necessary one, is the separation of the margins of the incisions in the integuments of the cheeks, bordering on the large cavities in the face, to the distance of a fourth or a third of an inch. It is by means of this separation that a firm attachment is procured for the skin forming the sides of the new nose, with the facial bones at its base, and consequently the nose is prevented from again sinking down by a sort of slipping outwards of its sides, especially at the upper portion. This mutual approximation of the sides, which will be again spoken of, is principally accomplished by means of two long needles, which are passed through the edges of the integuments of the face, under the base of the nose, and fixed, in a way to be afterwards described, to a long splint of stiff leather placed on each side of the nose, so as to squeeze it outwards by pressing on its base, and thus to push it prominently forward.

"Now begins the re-construction of the nose. If the surgeon were to bring the flaps in contact with the surface of the incised wounds, the nose certainly would not again sink, but it would be very flat. Their edges must therefore be cut in such a manner as to promote the upright position of the nose. For this purpose the inner part of the two edges of the dorsal flap is to be removed with a pair of sharp scissors, without comprehending in the incision any of the cuticle of its outer surface; so that the long thin strip thus cut away is of a triangular form. The reason for this is obvious. The dorsum of the nose acquires, in consequence, the properties of the key-stone of an arch, that is, it is between, and rests on, the edges of the external flaps. In order to prevent the strong inclination of the lateral flaps and alæ nasi inwards, and to raise them up like walls, their external edges too must be cut, not, however, on the inner angle of the edge, as in the instance of the dorsal flap, but on the outer or epidermal angle, from which about a straw's breadth is to be removed. On the straight lateral edges this is done with a pair of straight scissors, on the edges of the alæ with a pair of scissors curved on their surface.

"We now proceed to the most agreeable part of the operation, the union of the flaps with one another, and with the skin of the cheeks. In the first place, the edges of the dorsal flap, after the careful removal of the blood, are united with the upper or inner edges of the lateral flaps. The best mode of accomplishing this is by the twisted suture. Three needles are sufficient for each of the upper seams, and they must be placed at proper distances from each other; the two lowest should be at the sides of the tip of the nose. The union is more complete when another needle is introduced into the outermost edge of each ala. Finally, a ligature is to be passed through the edges of that part of the upper lip from which the septum was taken. This ligature therefore lies behind the septum; and, while it brings the edges of the breach in the lip closely together, it contributes to push the strip of integuments forward, and to prevent it from sinking back into its former cavity. All the needles, after being properly furnished with ligatures, should be cut off close to the thread. If the columna is altogether wanting, it is to be formed at a later period, after the parts are fully healed, by cutting a small strip of skin out of the upper lip, and bringing it in contact with the tip of the nose, where a raw surface is previously to be made for its reception.*

" One can scarcely imagine what firmness the nose has now acquired, although it still remains unattached at its lower part. The union of the nose with each of the basement edges of the cheek is effected by four simple knotted sutures, which are introduced by fine semicircular sharp needles. The two uppermost serve to fasten the lateral surfaces; the two lower to fix the alæ to the upper lip. The last and only remaining painful part of the operation which the patient has to undergo, is the insertion of two long needles under the nose, and through the detached edges of the integuments of the cheek, as has already been alluded to. The best method of effecting this, and the most convenient to the operator, is to thrust them from the left towards the right side; the distance from the point at which the pin enters to that at which it comes out, is upon an average about one inch. Before they are introduced a strip of stiff leather, from a third to half an inch in breadth, and from one and a half to two inches long, is laid on each side of the nose, forming two splints, which press the two sides of the nose together, keeping them in their proper situation. Each of these compressing pieces of leather has two small holes through which the needles are to be passed, on the left side before, but on the right side after, they have transfixed the nose. The heads of the needles on the left side

* "This method of renewing the columna nasi, when it alone is deficient, giving rise to a sinking of the tip and alæ of the nostrils, and not unfrequently to an exposure of the cavity, almost as hideous as that which results from the loss of the whole nose, was had recourse to for the first time in Britain in 1830, by Mr. Liston. It is remarkable, in such cases, that the mucous surface of the reflected portion of the lip very soon acquires all the properties of the dermoid tissue; while the dermoid surface degenerates into a kind of mucous membrane; the hairs even, previously constituting, if the subject be an adult male, a part of the beard, acquiring the character of those natural to the interior of the nostrils. Similar attempts to form a new columna had previously been made by Dupuytren of Paris, and Gersoul of Lyons; but both failed, probably from their having employed only the integuments of the lip, and not the whole substance of that organ." See *Introduction*, p. 44.—TRANSLATOR.

prevent them from slipping through the leather; which is also prevented on the right side and at the base of the nose, which at the same time is squeezed inwards to the requisite degree, by twisting the projecting points of the needles spirally with a pair of pliers.

"In conclusion, the nose, especially its cavities, must be carefully cleaned by injections of tepid water; and quills wrapped round with oiled charpie, are to be introduced into the nostrils.

"It is surely unnecessary to enter more fully into the details of this operation. Should the surgeon find my description not sufficiently circumstantial, and be unable to supply any thing from his own knowledge of general principles, that he may find wanting, he had better altogether abstain from operating." (P. 55.)

In the after-treatment, cold should never be employed as a means of reducing inordinate inflammation. Warm fomentations are to be employed soon after the operation, and continued until the inflammatory process be set up: when this process is established, a weak solution of the acetate of lead may be substituted. On the third day some of the needles may be removed, and a cautious attempt be made to withdraw the quills from the nostrils, which are to be gently cleansed by injections, and other quills substituted for those removed; these being wrapped in oiled lint previous to insertion, and changed daily. When the *septum* is destroyed, one large quill is to be used. The two long needles thrust through the base of the nose are to be removed on the eighth day, and two others introduced in different places, and left for eight days more. This may be again repeated. The splints are to be reapplied each time. The restoration being complete, the protection of the stranger is still a matter of interest and difficulty; for the adopted is in great peril of perishing from sheer kindness; and the warmth essential to the induction of the nose may after a time lead to its dispersion; just as Captain Ross was driven from his lodgings by the excess of temperature of this present November.

"In the after-treatment of the newly-formed nose, cold, in a greater or less degree, is preferable to all other applications. Observation has convinced me that the decay and death of the flap generally proceed from its being gorged with blood, which flowing in copiously, and unable to find egress, or to be returned by the veins, causes great distension, and subsequent mortification. In forming the bridge, care must be taken to destroy any artery that may present itself, and thus, as much as possible, to prevent any considerable quantity of blood being thrown into the flap. When this is done, for some time after the operation the new nose appears pale and withered; whereas, when it is omitted, it almost invariably becomes highly coloured, swells violently, and generally perishes from repletion. When such an accumulation of blood takes place, we must encourage a gentle flow from the lower edges of the flap. In no case should we tie a bleeding artery: if too much blood be lost, pressure must be employed, as then we are able to renew the bleeding as circumstances may require." (P. 151.)

The reader is referred to Gräfe's work on Rhinoplastics, for a more detailed account of the after-treatment.

To facilitate the removal of tumours from the nose, Dr. Dieffenbach directs to be done what is not less useful that it is rather oddly expressed: "I split up the alæ, and am thus easily enabled to get at the tumour." The doctor is not aware that this practice has been adopted by any one but himself in modern times; but he admits that it is spoken of by Hippocrates. "Should both nostrils be affected, I not only divide the alæ, but also the columna and septum, and turn back the nose during the operation. The wounds are readily united by the twisted suture, and generally heal in a few days."

The profession is greatly indebted to the author and translator: the book will be interesting to all, and useful to many; and the aforesaid obligation would be increased if the price of the volume were less. Of the plates we may truly say, with Goldsmith, that the picture would have been better if the painter had taken more pains.

